# Dietary factors and Alzheimer’s disease risk: a Mendelian randomization study

**DOI:** 10.1186/s40001-024-01821-8

**Published:** 2024-05-02

**Authors:** Qi Meng, Chen Chen, Mingfang Zhu, Yue Huang

**Affiliations:** Department of Neurology, Henan Provincial People’s Hospital, Zhengzhou University People’s Hospital, 7 Weiwu Street, Zhengzhou, 450000 China

**Keywords:** Alzheimer’s disease, Mendelian randomization, Dietary habits, Genome-wide association study

## Abstract

**Background:**

Prior observational research has investigated the association between dietary patterns and Alzheimer’s disease (AD) risk. Nevertheless, due to constraints in past observational studies, establishing a causal link between dietary habits and AD remains challenging.

**Methods:**

Methodology involved the utilization of extensive cohorts sourced from publicly accessible genome-wide association study (GWAS) datasets of European descent for conducting Mendelian randomization (MR) analyses. The principal analytical technique utilized was the inverse-variance weighted (IVW) method.

**Results:**

The MR analysis conducted in this study found no statistically significant causal association between 20 dietary habits and the risk of AD (All *p* > 0.05). These results were consistent across various MR methods employed, including MR-Egger, weighted median, simple mode, and weighted mode approaches. Moreover, there was no evidence of horizontal pleiotropy detected (All *p* > 0.05).

**Conclusion:**

In this MR analysis, our finding did not provide evidence to support the causal genetic relationships between dietary habits and AD risk.

**Supplementary Information:**

The online version contains supplementary material available at 10.1186/s40001-024-01821-8.

## Introduction

The aging global population has led to dementia emerging as a significant public health concern [1, 2]. According to the World Health Organization (WHO), the global prevalence of dementia was approximately 47 million in 2015, and it is projected to exceed 75 million by 2030 [3, 4]. Dementia significantly impacts both the physical and mental well-being of individuals, diminishes their quality of life, and imposes substantial pressure and financial strain on society and families [5]. It is noteworthy that Alzheimer’s disease (AD) stands as the most prevalent neurodegenerative form of dementia [6].

Numerous empirical studies have investigated the potential contributions of immune inflammation, mitochondrial dysfunction, genetic heredity, gut microbiota abnormalities, and cerebrovascular dysfunction to the pathogenesis of AD [7–9]. However, the exact etiology of AD remains unclear. Current pharmacological treatments for AD focus on symptom management without altering the disease progression [10]. Consequently, non-pharmacological interventions are being investigated to ameliorate symptoms and associated dysfunctions in AD.

Dietary interventions have emerged as a key area of research aimed at potentially slowing the onset and progression of AD [11, 12]. Specifically, the Mediterranean diet has been associated with a reduced risk of AD development [11], while a pro-inflammatory diet has been linked to an increased risk of AD [13, 14]. It is important to note that observational studies cannot establish direct causation, and a consensus on the influence of dietary habits on AD is lacking. Large-scale population-based studies are needed to provide genetic evidence supporting the potential impact of dietary interventions in reducing AD risk. Recognizing the potential benefits of dietary interventions for AD patients is clinically significant and requires further investigation in this area.

Mendelian randomization (MR) analysis is a statistical technique that employs genetic variants as instrumental variables to explore causal relationships between exposure factors and outcomes [15]. This method effectively utilizes results from genome-wide association studies (GWAS) to investigate the causal link between exposures and outcomes using genetic variants as instrumental variables (IVs) [16, 17]. Currently, there is a lack of comprehensive research on the causal relationship between dietary habits and AD at both national and international levels. Therefore, this study aimed to investigate the causal association between dietary habits and AD using a two-sample MR approach, aiming to offer valuable insights into this relationship and potentially informing new strategies for preventing and intervening in clinical diseases associated with AD.

## Materials and methods

### Study design and MR assumptions

**Fig. 1:**
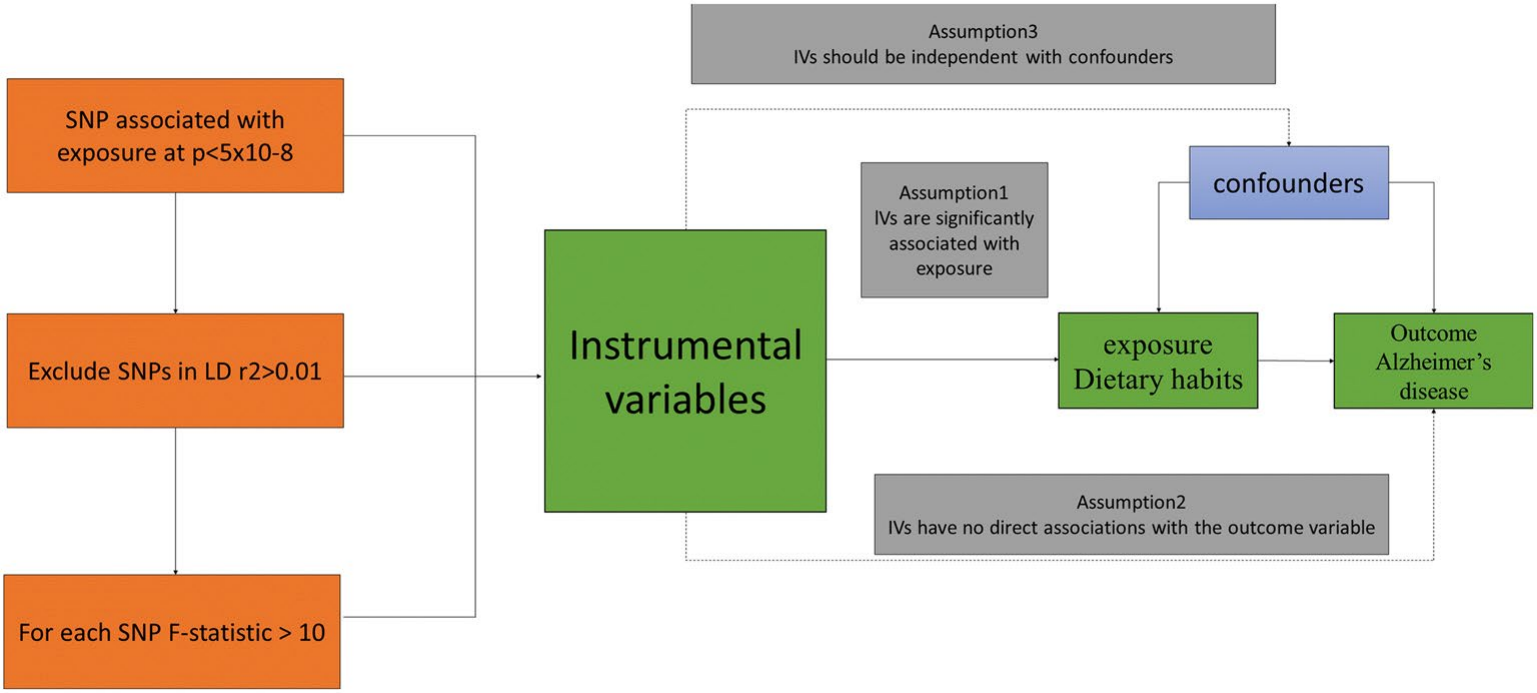
The central framework of Mendelian randomization analyses of the causal relationship of 20 dietary habits on the risk of AD. Assumption 1: IVs directly affect exposure; Assumption 2: IVs are not associated with confounders; Assumption 3: IVs influence risk of the outcome directly through the exposure

Figure 1 illustrates the study design. We examined bidirectional associations between dietary habits and AD using MR studies. We applied three key assumptions to genetic variants [18]: (1) SNPs are closely linked with exposure; (2) SNPs are not influenced by confounders along the exposure-outcome pathway; and (3) SNPs affect the outcome solely through exposure, without impacting the outcome through other pathways [19].

### Data sources

The genome-wide association data for the 20 dietary habits analyzed in this study were sourced from the UK Biobank (UKBB) GWAS summary statistics provided by the Benjamin Neale Laboratory (http://www.nealelab.is/uk-biobank/) [20]. The dataset analyzed 361,194 participants and included 13.7 million QC-passing SNPs [21]. Detailed information regarding the assessment questions for dietary habits is provided in Additional file 1: Table S1.

To identify genetic variants associated with AD prevalence, we utilized meta-analysis data from the IGAP [22]. This dataset comprised 63,926 subjects, including 21,982 AD cases and 41,944 healthy controls of European origin. Detailed information on all GWAS datasets is provided in Table 1.

**Table 1:** Basic information of the GWAS datasets used for the study

Traits	IEU GWAS id	Identified SNPs	Sample size	Population
Alzheimer’s Disease	ieu-b-2	NA	21,982/41,944	European
Water intake	ukb-b-14898	36	427,588	European
Salt added to food	ukb-b-8121	86	462,630	European
Dried fruit intake	ukb-b-16576	35	421,764	European
Coffee intake	ukb-b-5237	34	428,860	European
Salad / raw vegetable intake	ukb-b-1996	11	435,435	European
Cereal intake	ukb-b-15926	34	441,640	European
Fresh fruit intake	ukb-b-3881	49	446,462	European
Tea intake	ukb-b-6066	32	447,485	European
Cooked vegetable intake	ukb-b-8089	15	448,651	European
Cheese intake	ukb-b-1489	51	451,486	European
Bread intake	ukb-b-11348	25	452,236	European
Lamb/mutton intake	ukb-b-14179	26	460,006	European
Pork intake	ukb-b-5640	10	460,162	European
Oily fish intake	ukb-b-2209	46	460,443	European
Non-oily fish intake	ukb-b-17627	11	460,880	European
Beef intake	ukb-b-2862	12	461,053	European
Poultry intake	ukb-b-8006	7	461,900	European
Processed meat intake	ukb-b-6324	19	461,981	European
Hot drink temperature	ukb-b-14203	55	457,873	European
Alcohol intake frequency	ukb-b-5779	89	462,346	European
Alcoholic drinks per week	ieu-b-73	30	335,394	European

### Instrumental variable selection

Following the core assumptions of MR studies, SNPs with correlations meeting *p* < 5 × 10^–8^ were included as instrumental variables after screening the GWAS data. To minimize the influence of linkage disequilibrium (LD) on analysis outcomes, we imposed the condition of r^2^ < 0.001 and a window size of 10,000 kb [23, 24]; To ensure robust associations between instrumental and endogenous variables and to prevent weak instrumental variable bias, we calculated R^2^ [R^2^ = 2 × EAF × (1 − EAF) × b^2^], representing the proportion of variation explained by instrumental variable SNPs, and the F statistic [F = R^2^ × (N − 2)/(1 − R^2^)], used to evaluate the strength of instrumental variables, for each SNP separately.

### Statistical analysis

The IVW analysis, a meta-analysis of the Wald ratios for each SNP using inverse variance weighting, is utilized to obtain an MR estimate [25]. MR-Egger regression, a weighted linear regression of effect estimates for exposure and outcome, differs from the IVW method in that it imposes no restriction on the intercept and permits all SNPs in the MR analysis to be potentially invalid IVs [26]. The Egger intercept enables the detection of horizontal pleiotropy among SNPs in MR analyses [27].

Gene pleiotropy was assessed using the intercept of MR-Egger regression, where larger values indicate a higher likelihood of pleiotropy. If the pleiotropy test yielded a *p*-value > 0.05, its effect on causal estimation was disregarded. MR-PRESSO examined multiple SNP studies for outliers and pleiotropy, with a *p*-value > 0.05 indicating no pleiotropy [28]. Lastly, sensitivity analysis was conducted using the Leave-one-out method to assess the individual SNP effects on the results [29]. Differences were considered statistically significant at *p* < 0.05 in each analysis, and assays were bidirectional and used the Two Sample MR [30], MR-PRESSO [28] and Mendelian Randomization [31] packages in the R software (version 4.0.2).

## Results

### Causal relationship between dietary habits and AD

In the MR analysis, after excluding palindromic SNPs and SNPs related to confounding factors, the numbers of SNPs that ultimately identified as the IVs for different dietary habits in the MR analysis were 36 (Water intake), 34 (Cereal intake), 86 (Salt added to food), 35 (Dried fruit intake), 34 (Coffee intake), 51 (Cheese intake), 11 (Salad/raw vegetable intake), 49 (Fresh fruit intake), 32 (Tea intake), 15 (Cooked vegetable intake), 10 (Pork intake), 25 (Bread intake), 26 (Lamb/mutton intake), 46 (Oily fish intake), 7(Poultry intake),11 (Non-oily fish intake), 12 (Beef intake),19 (Processed meat intake), 55 (Hot drink temperature), 89 (Alcohol intake frequency), and 30 (Alcoholic drinks per week). The F-statistics were all greater than 10, indicating no weak instrumental bias (Table 1).

**Fig. 2:**
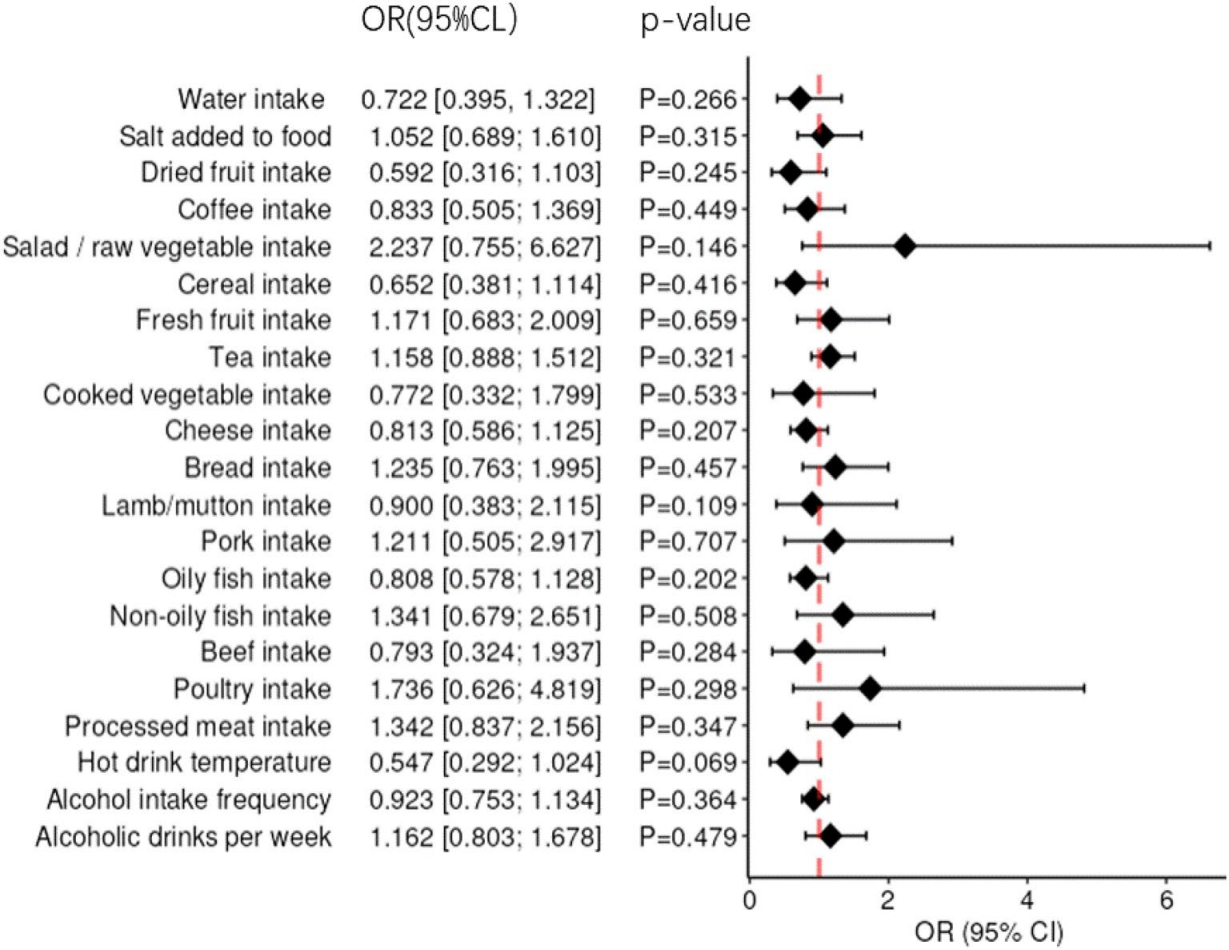
Summary of the MR estimation in IVW. *OR* odds ratio, *CI* confidence interval, *p* *p* value of MR

In the MR analysis, we found that water intake (OR = 0.722 95%CI: 0.395–1.322, *p* = 0.266), Salt added to food (OR = 1.052, 95%CI: 0.689–1.610, *p* = 0.315), Dried fruit intake (OR = 0.592, 95%CI: 0.316–1.103, *p* = 0.245), Coffee intake (OR = 0.833, 95%CI: 0.505–1.369, *p* = 0.449), Salad/raw vegetable intake (OR = 2.237, 95%CI: 0.755–6.627, *p* = 0.146), Cereal intake (OR = 0.652, 95%CI: 0.381–1.114, *p* = 0.416), Fresh fruit intake (OR = 1.171, 95%CI: 0.683–2.009, *p* = 0.659), Tea intake (OR = 1.158, 95%CI: 0.888–1.512, *p* = 0.321), Cooked vegetable intake (OR = 0.772, 95%CI: 0.332–1.799, *p* = 0.533), Cheese intake (OR = 0.813, 95%CI: 0.586–1.125, *p* = 0.207), Bread intake (OR = 1.235, 95%CI: 0.763–1.995, *p* = 0.457), Lamb/mutton intake (OR = 0.900, 95%CI: 0.383–2.115, *p* = 0.109), Pork intake(OR = 1.211, 95%CI: 0.505–2.917, *p* = 0.707), Oily fish intake (OR = 0.808, 95%CI: 0.578–1.128, *p* = 0.202), Non-oily fish intake (OR = 1.341, 95%CI: 0.679–2.651, *p* = 0.508), Processed meat intake (OR = 1.342, 95%CI: 0.837–2.156, *p* = 0.347), Hot drink temperature (OR = 0.547, 95%CI: 0.292–1.024, *p* = 0.069), Beef intake (OR = 0.793, 95%CI: 0.324–1.937, *p* = 0.284), Poultry intake(OR = 1.736, 95%CI: 0.626–4.819, *p* = 0.298), Alcohol intake frequency (OR = 0.923, 95%CI: 0.753–1.134, *p* = 0.364), Alcoholic drinks per week (OR = 1.162, 95%CI: 0.803–1.678, *p* = 0.479) were not associated with AD risk. To sum up, there was no significant causal relationship between 20 dietary habits and risk of AD (All *p* > 0.05). Furthermore, the IVW results of the MR analysis are shown in Fig. 2. And are illustrated as a scatter plot (Additional file 1: Figure S1).

### Sensitivity analyses

The Cochran’s Q test identified heterogeneity among the IVs related to dried fruit intake, coffee intake, cereal intake, fresh fruit intake, bread intake, hot drink temperature, alcohol intake frequency, and alcoholic drinks per week (refer to Table 2). Notably, there was no significant evidence of horizontal pleiotropy observed in the correlation between dietary habits and AD, as all p-values exceeded 0.05. This enhances the reliability of the inferred causal relationships based on our findings. Moreover, a leave-one-out sensitivity analysis was performed to evaluate the impact of individual SNPs on the causal effect, confirming that no single SNP was driving the observed effect (see Additional file 1: Figure S2). Additionally, a funnel plot was employed for visualization purposes (refer to Additional file 1: Figure S3).

**Table 2:** The results of heterogeneity and horizontal pleiotropy tests

Traits	Heterogeneity test		Horizontal pleiotropy test
	MR-Egger regression	IVW model	MR-Egger intercept
Water intake	0.038	0.030	0.998
Salt added to food	0.211	0.191	0.958
Dried fruit intake	< 0.001	< 0.001	0.879
Coffee intake	< 0.001	< 0.001	0.269
Salad / raw vegetable intake	0.265	0.320	0.229
Cereal intake	0.045	0.040	0.496
Fresh fruit intake	< 0.001	< 0.001	0.410
Tea intake	0.727	0.705	0.496
Cooked vegetable intake	0.321	0.443	0.118
Cheese intake	0.068	0.059	0.644
Bread intake	0.034	0.025	0.827
Lamb/mutton intake	0.200	0.196	0.373
Pork intake	0.776	0.715	0.634
Oily fish intake	0.669	0.644	0.557
Non-oily fish intake	0.282	0.483	0.093
Beef intake	0.205	0.172	0.580
Poultry intake	0.455	0.425	0.413
Processed meat intake	0.103	0.109	0.325
Hot drink temperature	< 0.001	< 0.001	0.863
Alcohol intake frequency	< 0.001	< 0.001	0.413
Alcoholic drinks per week	< 0.001	< 0.001	0.466

## Discussion

There has been increasing attention to the relationship between healthy eating habits and neurodegenerative diseases, particularly AD [32]. Numerous studies have investigated the impact of nutrients and dietary patterns on AD prevention [33]. Specifically, epidemiological evidence suggests that individuals consuming a pro-inflammatory diet have a higher risk of developing AD [34, 35], while adherence to the Mediterranean diet is associated with a reduced risk of AD [36]. Additionally, a meta-analysis of 11 observational studies has shown that increased fish consumption may benefit AD patients [37]. Conversely, a dietary pattern characterized by relatively high carbohydrate intake has been linked to an increased risk of AD in older adults [38]. Generally, dietary factors are believed to potentially influence the risk of cognitive decline (CD) and AD through endogenous mechanisms triggered by the food metabolome (Additional files 2, 3).

The consistency of findings regarding the relationship between diet and AD risk varies. A prospective cohort study involving 8225 participants without dementia revealed that diet quality during midlife was not significantly associated with subsequent dementia risk [39]. Similarly, another prospective study with 2232 individuals and a mean follow-up of 6.9 years found no significant association between diet quality and the risk of AD and other forms of dementia [40]. Furthermore, a meta-analysis of 5 randomized controlled trials (RCTs) reported significant heterogeneity in the association between dietary habits and AD risk [41]. In our MR study, we did not find any statistically significant causal association between dietary habits and AD, which contradicts some prior research findings [42–44]. Therefore, establishing causality necessitates additional controlled trials.

There are various possible explanations for this disparity in the results. While MR can effectively address bias caused by confounding factors, it does not replace RCTs and serves as a valuable complement to them [45, 46]. Therefore, caution must be exercised when interpreting this conclusion. Most findings in this area originate from epidemiological studies, which, despite demonstrating correlations between dietary habits and AD, do not establish causality. Observational studies may be influenced by confounding factors such as socio-economic status, lifestyle habits, and physical health [47]. Moreover, discrepancies in data sources across different MR studies may also contribute to the inconsistency. For instance, previous cohort studies reported no association between coffee intake and AD incidence [48], consistent with earlier MR studies [49, 50]. However, recent research has shown a protective effect of coffee intake against neurodegenerative diseases, particularly AD [51]. Additionally, previous studies have highlighted the gender-related aspect of beverages consumption and cognitive impairment [32]. Therefore, further investigation into gender differences in diet and neuroprotection is warranted [36].

AD is a highly complex disease influenced by both genetic and environmental factors, and the exact role of nutrients in its pathogenesis remains unclear. Mechanisms underlying the association between dietary habits and changes in AD risk have not been fully elucidated in previous studies [44]. Epigenetic, gut microbiome, and brain imaging studies could shed light on these mechanisms and warrant further investigation. While no causal association between dietary habits and AD was found in our study, this does not negate the possibility that dietary preferences could hasten cognitive decline in AD patients. Evidence suggests that chronic neuroinflammation plays a crucial role in AD pathogenesis [13], with peripheral inflammatory responses linked to AD pathology [52]. A recent prospective study on the dietary inflammatory index (DII) indicated that increased DII may elevate the risk of AD [HR: 1.391, 95% CI: 1.085–1.784] [13]. Thus, specific dietary interventions may exacerbate AD through the mediation of neuroinflammation rather than dietary habits alone.

This study provides valuable insights into the causal relationship between dietary habits and AD risk, but caution is needed in interpreting the causal evidence due to the need for further validation. Several limitations need to be acknowledged. Firstly, the study was limited to individuals of European ancestry, potentially affecting the generalizability of the findings. Future research should include diverse populations to confirm the results. Secondly, the study focused solely on dietary habits and did not consider principal component (PC) analyses conducted by Cole et al. [53]. Thirdly, due to data limitations, specific dietary patterns’ effects on dementia risk could not be explored. Moreover, using GWAS data from multiple consortia may introduce heterogeneity into the analysis. Efforts to address pleiotropy in the MR study may not have eliminated all instances, potentially biasing the results. Additionally, effect sizes and dose–response relationships could not be accurately estimated in the study.

## Conclusions

In summary, our MR analysis did not reveal any causal genetic associations between dietary habits and AD risk. However, due to the intricate interplay and limited research evidence on the pathophysiological mechanisms connecting AD and dietary patterns, further studies are warranted to validate our findings and elucidate potential mechanisms.

### Supplementary Information

**Additional file 1: Table S1.** Summary of 20 dietary habits questionnaire. **Table S2.** Results for Mendelian randomization analyses (IVW). **Figure S1.** Scatterplot analysis for dietary habits and AD. **Figure s2.** MR leave-one-out analysis for dietary habits and AD. **Figure S3.** Funnel plots of the association between dietary habits and AD.

**Additional file 2.** SNPS for dietary habits.

**Additional file 3.** The results of replication validation.

## Data Availability

The original contributions presented in the study are included in the article/Supplementary Material. The dataset generated during and analyzed during the current study are available from the MR Base database (http://www.mrbase.org/).
