# Supplementary material for: Dietary factors and Alzheimer’s disease risk: a Mendelian randomization study
Source: Eur J Med Res. 2024 May 2;29:261. doi: 10.1186/s40001-024-01821-8 (PMC11067192; doi:10.1186/s40001-024-01821-8)
Supplement: Supplementary file 1 — Additional file 1: Table S1. Summary of 20 dietary habits questionnaire. Table S2. Results for Mendelian randomization analyses (IVW). Figure S1. Scatterplot analysis for dietary habits and AD. Figure s2. MR leave-one-out analysis for dietary habits and AD. Figure S3. Funnel plots of the association between dietary habits and AD. [file 40001_2024_1821_MOESM1_ESM.zip › Supplementary 1/Supplement material Table S1 and S2.docx]

**Dietary Factors and Alzheimer’s Disease Risk: A Mendelian**

**Randomization Study**

**Supplementary Information 1**

**Table S1.** Summary of 20 dietary habits questionnaire

**Table S2.** Results for Mendelian randomization analyses (IVW)

**Figure S1.** Scatterplot analysis for dietary habits and AD.

**Figure s2.** MR leave-one-out analysis for dietary habits and AD.

**Figure S3.** Funnel plots of the association between dietary habits and AD

**Table S1** Summary of 20 dietary habits questionnaire

| **Trait** | **Question** | **Help button information** | **Notes** |
| --- | --- | --- | --- |
| Cooked vegetable intake | On average how many heaped tablespoons of COOKED vegetables would you eat per DAY? (Do not include potatoes; put '0' if you do not eat any) | Please provide an average considering your intake over the last year. If you are unsure, please provide an estimate or select Do not know. If you have less than one tablespoon a day select Less than one. | If answer > 50 then rejected -10 represents "Less than one" -1 represents "Do not know" -3 represents "Prefer not to answer" |
| Salad / raw vegetable intake | On average how many heaped tablespoons of SALAD or RAW vegetables would you eat per DAY? (Include lettuce, tomato in sandwiches; put '0' if you do not eat any) | Please provide an average considering your intake over the last year. If you are unsure, please provide an estimate or select Do not know. If you have less than one tablespoon a day select Less than one. | If answer > 50 then rejected -10 represents "Less than one" -1 represents "Do not know" -3 represents "Prefer not to answer" |
| Fresh fruit intake | About how many pieces of FRESH fruit would you eat per DAY? (Count one apple, one banana, 10 grapes etc as one piece; put '0' if you do not eat any) | Please provide an average considering your intake over the last year. If you are unsure, please provide an estimate or select Do not know | If answer > 50 then rejected -10 represents "Less than one" -1 represents "Do not know" -3 represents "Prefer not to answer" |
| Dried fruit intake | About how many pieces of DRIED fruit would you eat per DAY? (Count one prune, one dried apricot, 10 raisins as one piece; put '0' if you do not eat any) | Please provide an average considering your intake over the last year. If you are unsure, please provide an estimate or select Do not know. | If answer > 100 then rejected -10 represents "Less than one" -1 represents "Do not know" -3 represents "Prefer not to answer" |
| Oily fish intake | How often do you eat oily fish? (e.g. sardines, salmon, mackerel, herring) | Please provide an average considering your intake over the last year. If you are unsure, please provide an estimate or select Do not know. Oily fish include: Salmon Anchovies, Trout Swordfish, Mackerel Bloater, Herring Cacha, Sardines Carp, Pilchards Hilsa, Kipper Jack fish, Eel Katla, Whitebait Orange roughy, Tuna (fresh only) Pangas, Sprats | Options: never, less than once a week, once a week, 2-4 times a week, 5-6 times a week, once or more daily, do not know, prefer not to answer |
| Non-oily fish intake | How often do you eat other types of fish? (e.g. cod, tinned tuna, haddock) | Please provide an average considering your intake over the last year. If you are unsure, please provide an estimate or select Do not know. | Options: never, less than once a week, once a week, 2-4 times a week, 5-6 times a week, once or more daily, do not know, prefer not to answer |
| Processed meat intake | How often do you eat processed meats (such as bacon, ham, sausages, meat pies, kebabs, burgers, chicken nuggets)? | Please provide an average considering your intake over the last year If you are unsure, please provide an estimate or select Do not know. | Options: never, less than once a week, once a week, 2-4 times a week, 5-6 times a week, once or more daily, do not know, prefer not to answer |
| Poultry intake | How often do you eat chicken, turkey or other poultry? (Do not count processed meats) | Please provide an average considering your intake over the last year If you are unsure, please provide an estimate or select Do not know. | Options: never, less than once a week, once a week, 2-4 times a week, 5-6 times a week, once or more daily, do not know, prefer not to answer |
| Beef intake | How often do you eat beef? (Do not count processed meats) | Please provide an average considering your intake over the last year If you are unsure, please provide an estimate or select Do not know. | Options: never, less than once a week, once a week, 2-4 times a week, 5-6 times a week, once or more daily, do not know, prefer not to answer |
| Lamb/mutton intake | How often do you eat lamb/mutton? (Do not count processed meats) | Please provide an average considering your intake over the last year If you are unsure, please provide an estimate or select Do not know. | Options: never, less than once a week, once a week, 2-4 times a week, 5-6 times a week, once or more daily, do not know, prefer not to answer |
| Pork intake | How often do you eat pork? (Do not count processed meats such as bacon or ham) | Please provide an average considering your intake over the last year If you are unsure, please provide an estimate or select Do not know. | Options: never, less than once a week, once a week, 2-4 times a week, 5-6 times a week, once or more daily, do not know, prefer not to answer |
| Cheese intake | How often do you eat cheese? (Include cheese in pizzas, quiches, cheese sauce etc) | Please provide an average considering your intake over the last year If you are unsure, please provide an estimate or select Do not know. | Options: never, less than once a week, once a week, 2-4 times a week, 5-6 times a week, once or more daily, do not know, prefer not to answer |
| Bread intake | How many slices of bread do you eat each WEEK? | For other types of bread: - one bread roll = 2 slices - one pitta bread = 2 slices | If answer < 0 then rejected If answer > 250 then rejected If answer > 50 then participant asked to confirm -10 represents "Less than one" -1 represents "Do not know" -3 represents "Prefer not to answer" |
| Cereal intake | How many bowls of cereal do you eat a WEEK? | Please provide an average considering your intake over the last year. If you are unsure, please provide an estimate or select Do not know. | If answer < 0 then rejected If answer > 99 then rejected If answer > 14 then participant asked to confirm -10 represents "Less than one" -1 represents "Do not know" -3 represents "Prefer not to answer" |
| Salt added to food | Do you add salt to your food? (Do not include salt used in cooking) | Please provide an average considering your intake over the last year If you are unsure, please provide an estimate or select Do not know. | Options: never/ rarely, sometimes, usually, always, prefer not to answer |
| Tea intake | How many cups of tea do you drink each DAY? (Include black and green tea) | Please provide an average considering your intake over the last year. If you are unsure, please provide an estimate or select Do not know. | If answer < 0 then rejected If answer > 99 then rejected If answer > 20 then participant asked to confirm -10 represents "Less than one" -1 represents "Do not know" -3 represents "Prefer not to answer" |
| Coffee intake | How many cups of coffee do you drink each DAY? (Include decaffeinated coffee) | Please provide an average considering your intake over the last year. If you are unsure, please provide an estimate or select Do not know. | If answer < 0 then rejected If answer > 99 then rejected If answer > 10 then participant asked to confirm -10 represents "Less than one" -1 represents "Do not know" -3 represents "Prefer not to answer" |
| Hot drink temperature | How do you like your hot drinks? (Such as coffee or tea) | Not available | Options: very hot, hot, warm, do not drink hot drinks, prefer not to answer |
| Water intake | How many glasses of water do you drink each DAY? | Please provide an average considering your intake over the last year. If you are unsure, please provide an estimate or select Do not know. | If answer < 0 then rejected If answer > 99 then rejected If answer > 10 then participant asked to confirm -10 represents "Less than one" -1 represents "Do not know" -3 represents "Prefer not to answer" |
| Alcohol intake frequency | About how often do you drink alcohol? | If this varies a lot, please provide an average considering your intake over the last year | Options: daily or almost daily, three or four times a week, once or twice a week, one to three times a month, special occasions only, never, prefer not to answer |

Note: The information of the questionnaire was extracted from the UK biobank website.

**Table S2: Results for Mendelian randomization analyses (IVW)**

| **Traits** | **B** | **SE** | **p** |
| --- | --- | --- | --- |
| Water intake | -0.3230 | 0.2881 | 0.2661 |
| Salt added to food | 0.0506 | 0.1951 | 0.3145 |
| Dried fruit intake | -0.5238 | 0.3431 | 0.2451 |
| Coffee intake | -0.1834 | 0.2423 | 0.4489 |
| Salad / raw vegetable intake | 0.8051 | 0.5541 | 0.1462 |
| Cereal intake | -0.4284 | 0.3245 | 0.4160 |
| Fresh fruit intake | 0.1582 | 0.3581 | 0.6588 |
| Tea intake | 0.1474 | 0.1486 | 0.3213 |
| Cooked vegetable intake | -0.2583 | 0.5301 | 0.5327 |
| Cheese intake | -0.2077 | 0.1646 | 0.2072 |
| Bread intake | 0.2103 | 0.2828 | 0.4572 |
| Lamb/mutton intake | -0.1058 | 0.4932 | 0.1091 |
| Pork intake | 0.1918 | 0.5101 | 0.7068 |
| Oily fish intake | --0.2132 | 0.1671 | 0.2022 |
| Non-oily fish intake | 0.2940 | 0.4445 | 0.5084 |
| Beef intake | -0.2326 | 0.5470 | 0.2840 |
| Poultry intake | 0.5512 | 0.5296 | 0.2980 |
| Processed meat intake | 0.2941 | 0.3125 | 0.3465 |
| Hot drink temperature | -0.6028 | 0.3317 | 0.0691 |
| Alcohol intake frequency | -0.0801 | 0.0882 | 0.3638 |
| Alcoholic drinks per week | 0.1497 | 0.2112 | 0.4787 |
