# Supplementary figures and images for: Dietary factors and Alzheimer’s disease risk: a Mendelian randomization study
Source: Eur J Med Res. 2024 May 2;29:261. doi: 10.1186/s40001-024-01821-8 (PMC11067192; doi:10.1186/s40001-024-01821-8)

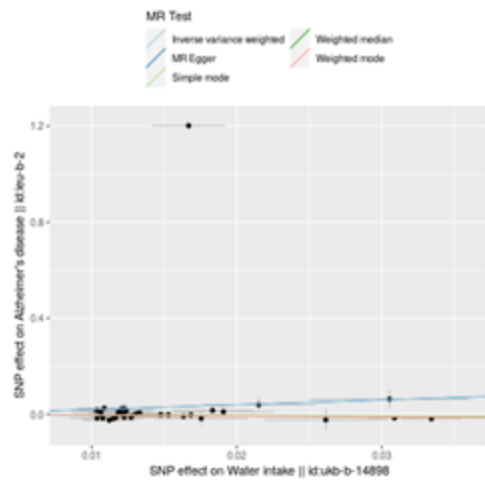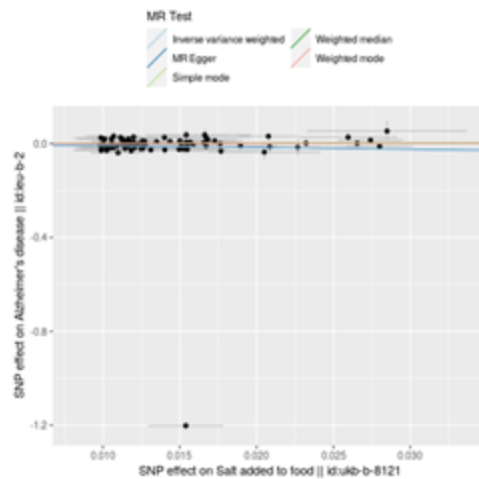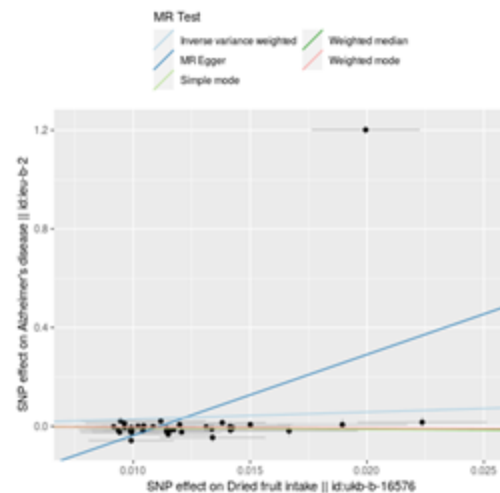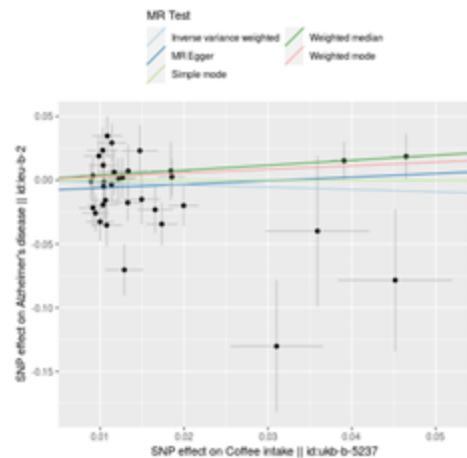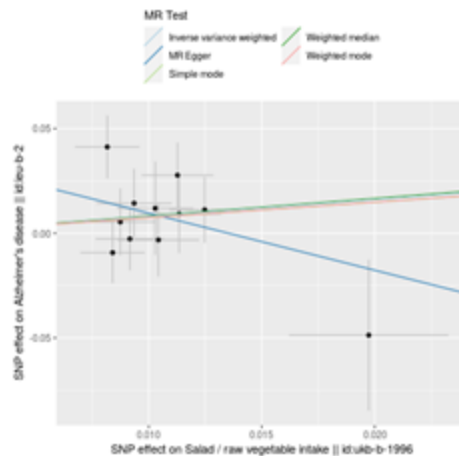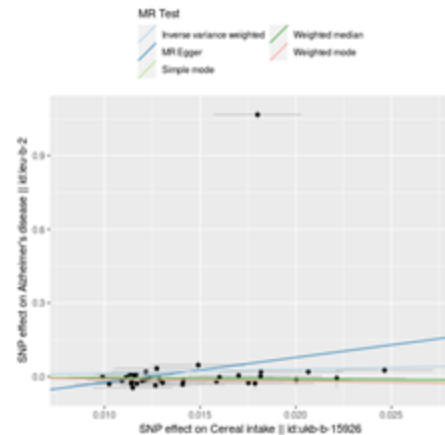

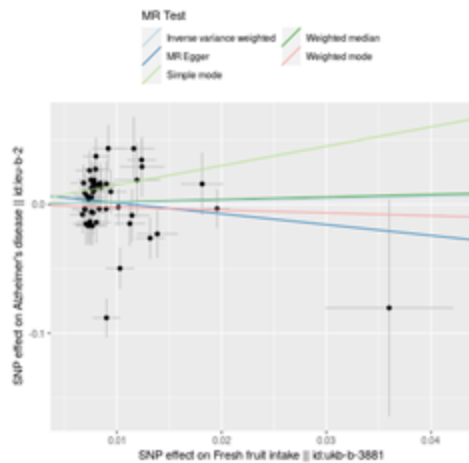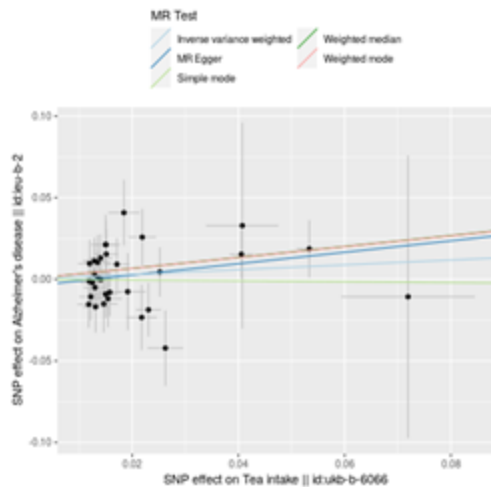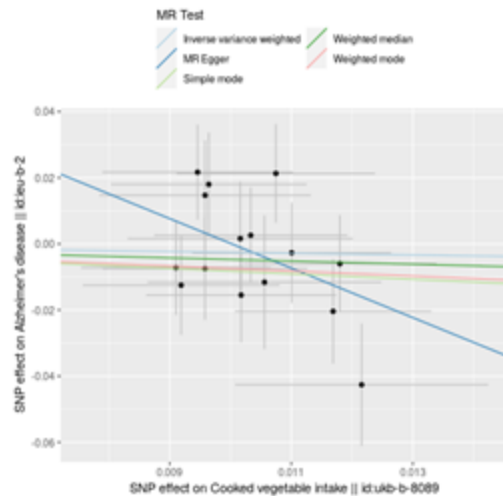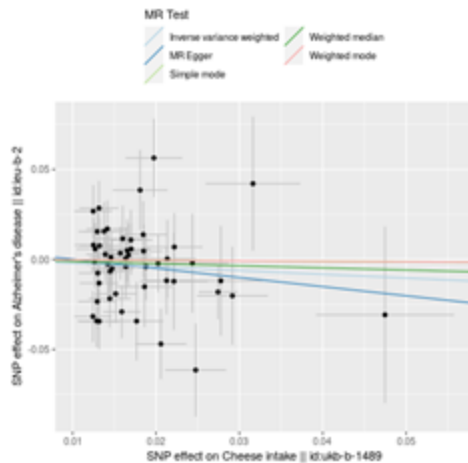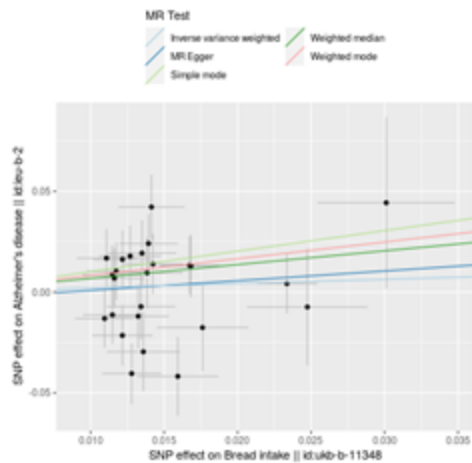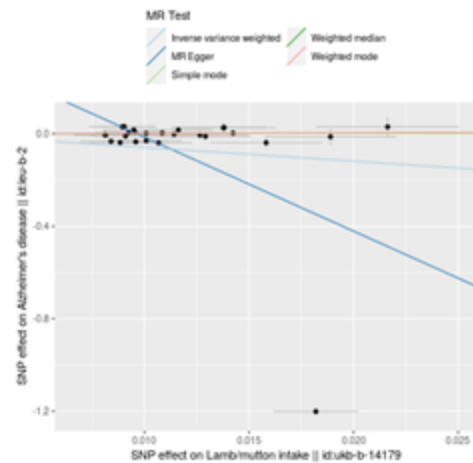

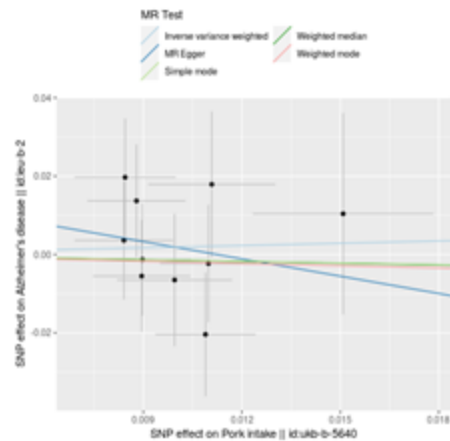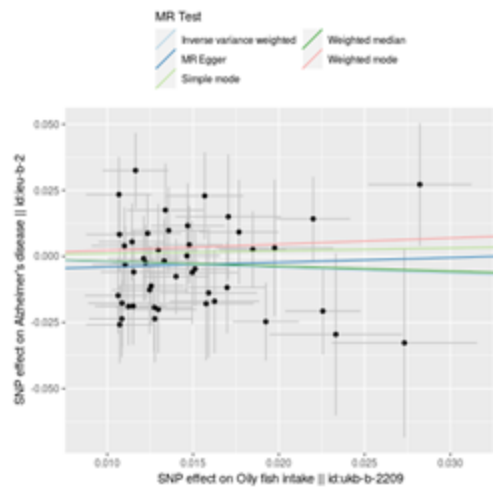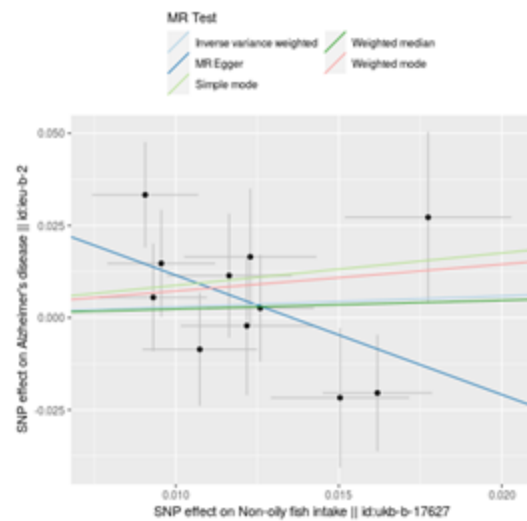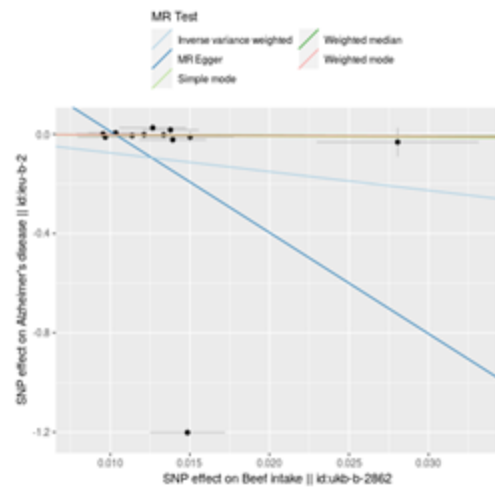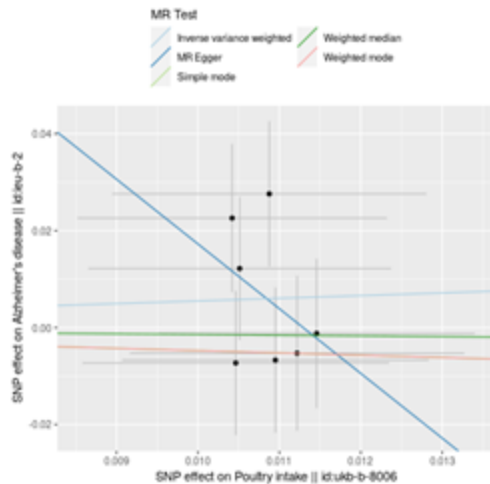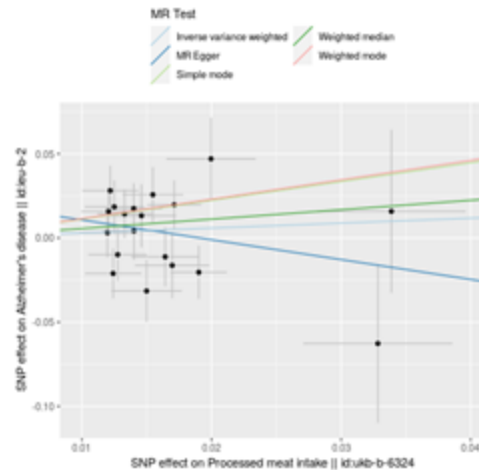

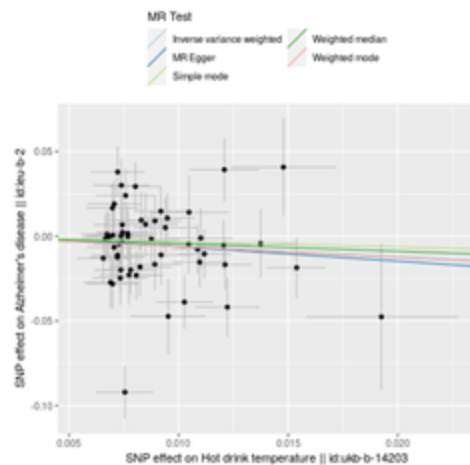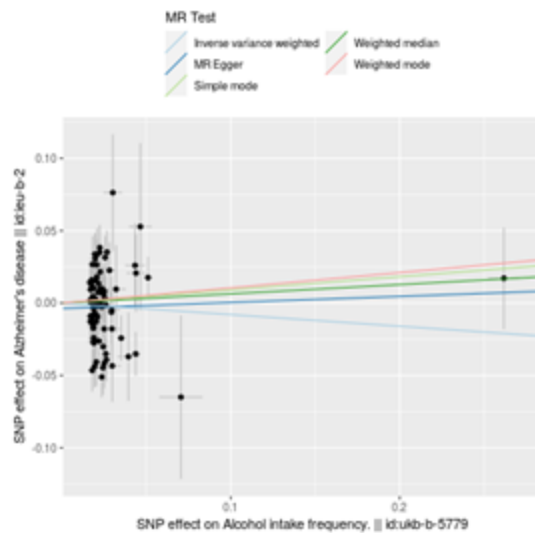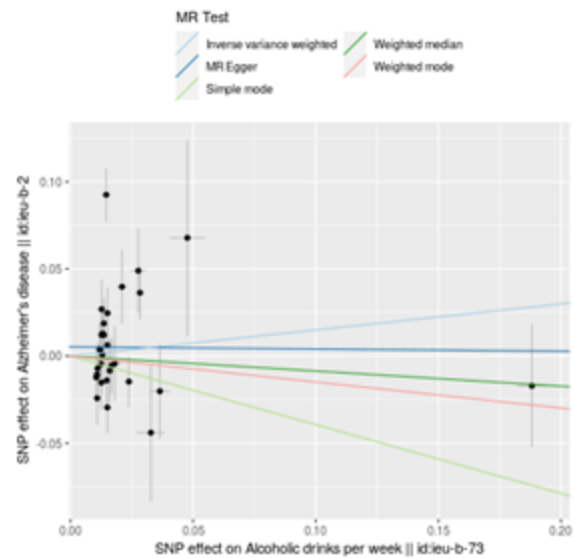

Supplement: Supplementary file 1 — Additional file 1: Table S1. Summary of 20 dietary habits questionnaire. Table S2. Results for Mendelian randomization analyses (IVW). Figure S1. Scatterplot analysis for dietary habits and AD. Figure s2. MR leave-one-out analysis for dietary habits and AD. Figure S3. Funnel plots of the association between dietary habits and AD. [file 40001_2024_1821_MOESM1_ESM.zip › Supplementary 1/Supplement material Figure S1]

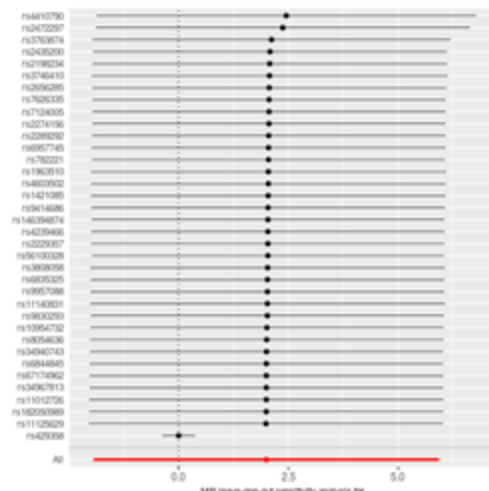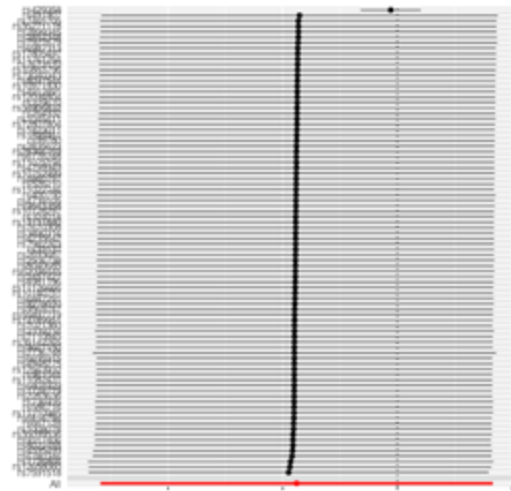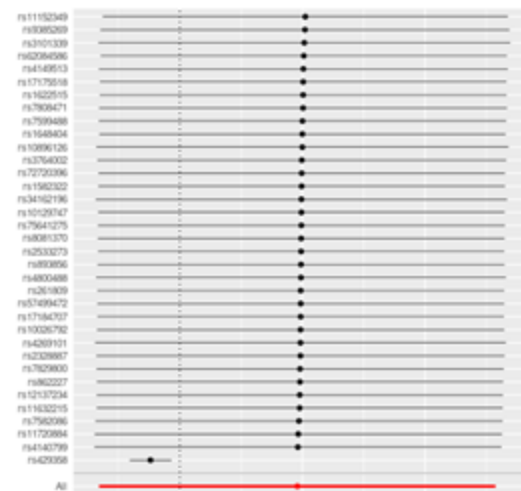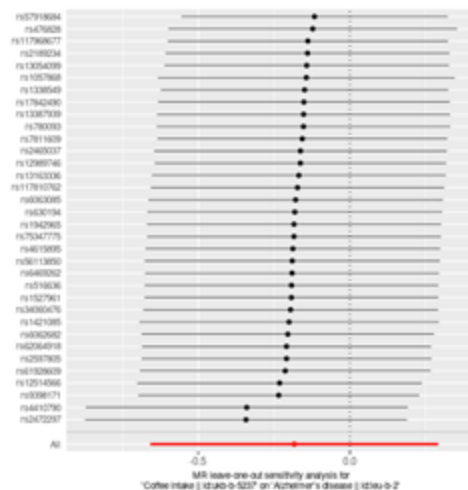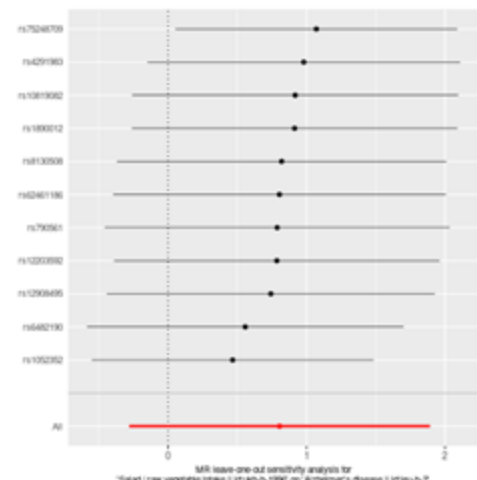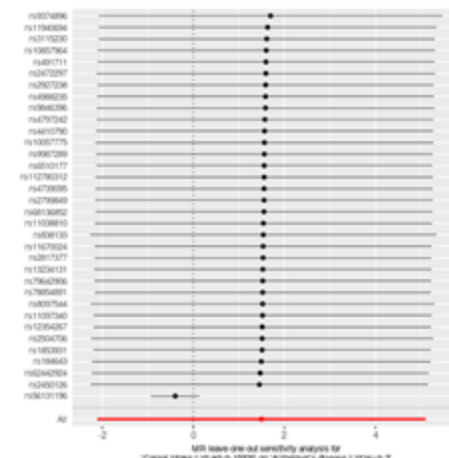

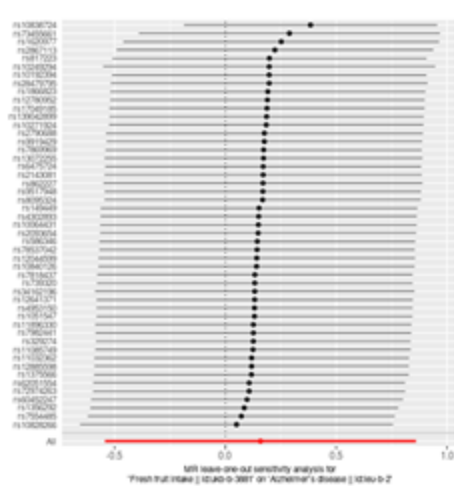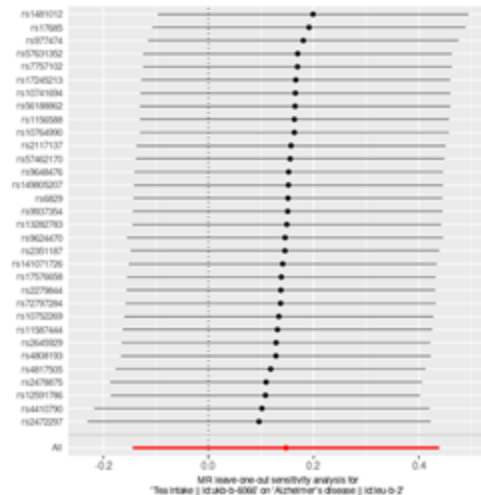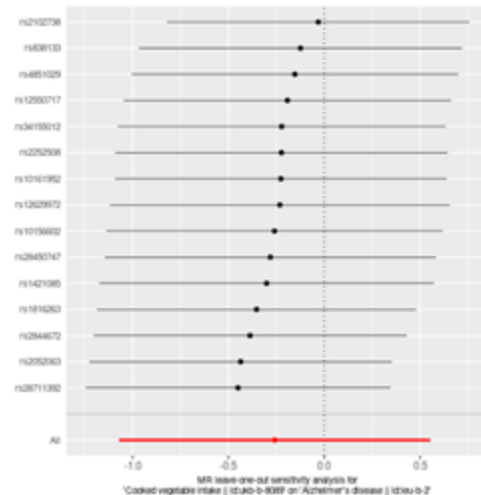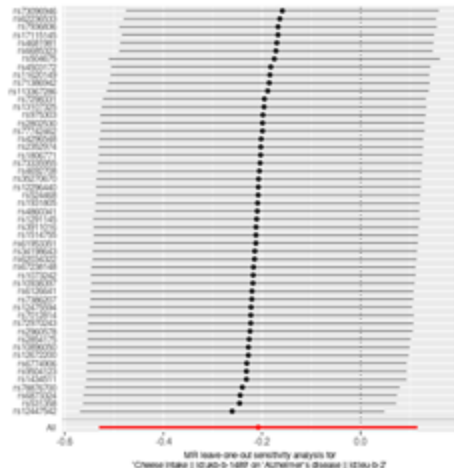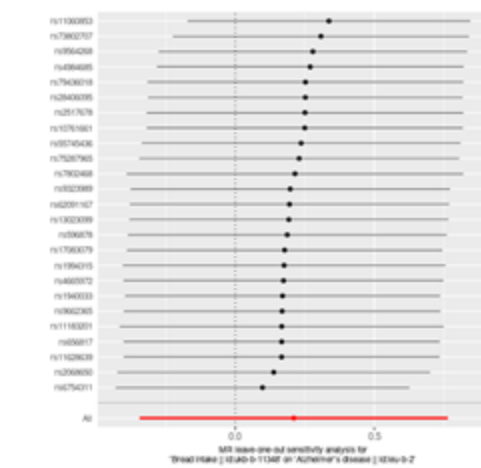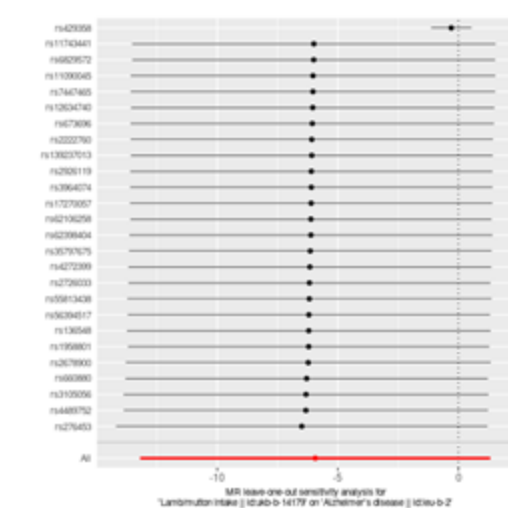

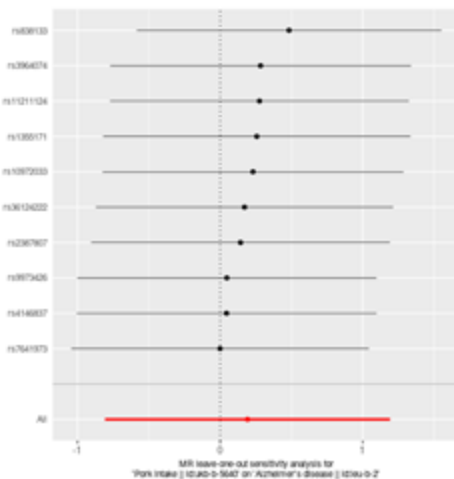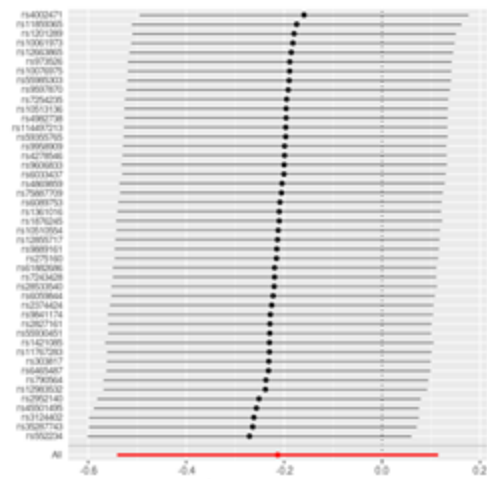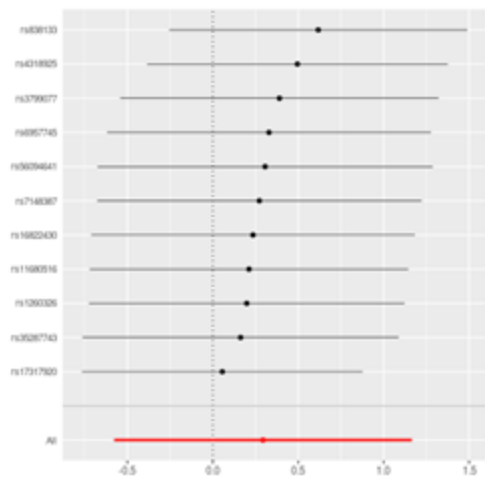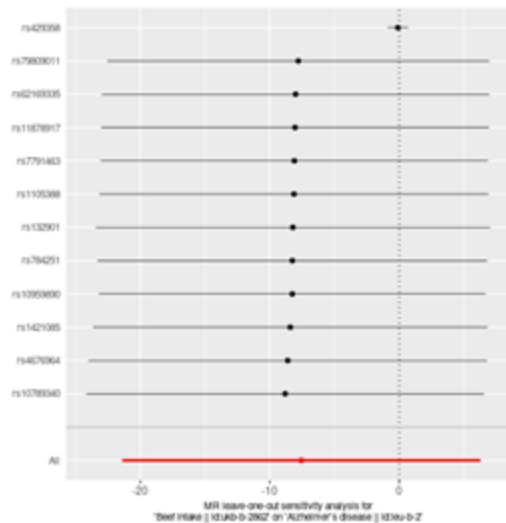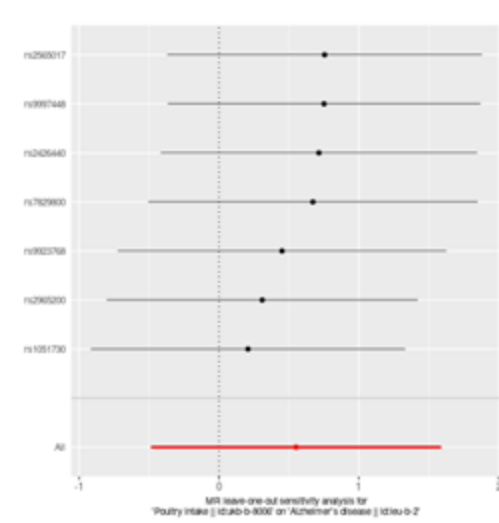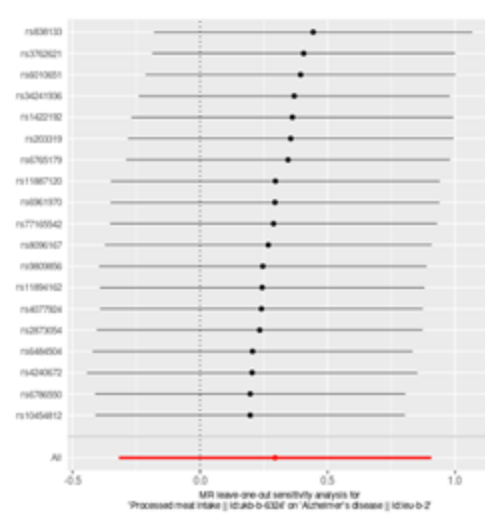

Supplement: Supplementary file 1 — Additional file 1: Table S1. Summary of 20 dietary habits questionnaire. Table S2. Results for Mendelian randomization analyses (IVW). Figure S1. Scatterplot analysis for dietary habits and AD. Figure s2. MR leave-one-out analysis for dietary habits and AD. Figure S3. Funnel plots of the association between dietary habits and AD. [file 40001_2024_1821_MOESM1_ESM.zip › Supplementary 1/Supplement material Figure s2]
